# Norovirus P particle-based tau vaccine-generated phosphorylated tau antibodies markedly ameliorate tau pathology and improve behavioral deficits in mouse model of Alzheimer’s disease

**DOI:** 10.1038/s41392-020-00416-z

**Published:** 2021-02-13

**Authors:** Yao Sun, Yongqing Guo, Xuejian Feng, Lu Fu, Yayuan Zheng, Yue Dong, Yong Zhang, Xianghui Yu, Wei Kong, Hui Wu

**Affiliations:** 1 National Engineering Laboratory for AIDS Vaccine, School of Life Sciences, Jilin University, Changchun, 130012 China; 2 Laboratory of Pathogenic Microbiology and Immunology, College of Life Science, Jilin Agricultural University, Changchun, 130012 China; 3 Key Laboratory for Molecular Enzymology and Engineering, the Ministry of Education, School of Life Sciences, Jilin University, Changchun, 130012 China

**Keywords:** Vaccines, Diseases of the nervous system

**Dear Editor,**

Currently, there are no FDA-approved disease-modifying therapies that can prevent, halt, or reverse Alzheimer’s disease (AD). As the unsatisfactory of amyloid-β-targeted treatment in recent years, development of Tau-targeted active immunotherapy takes much concern.^1^ Tau protein, a major microtubule-associated protein in the nervous system, was found to be abnormally hyperphosphorylated at six epitopes: Ser396/404, Ser202, Thr205, Ser238, and Ser262 in AD patients.^2^ Hence, immunotherapy targeting more highly-expressed phosphorylated Tau (pTau) species may induce a sufficient pool of pTau antibodies to eliminate pathological tau and elicit cognitive improvement. Although several prior immunotherapies targeting pTau, including pTauS202/T205, pT181, and pTauS396/S404, also appeared benefit to AD, there are a few limitations.^3^ First, the rationale for pTau epitope choice is based only on a phospho-epitope or is not stated. Second, the most effective treatment time is not determined. Finally, the mechanism of action of pTau antibody is not fully elucidated. Here we developed a potent Noroviruses (NoVs) P particle (PP)-based active immunotherapy targeting optimal pathological tau species, exploited its effectiveness in premorbid and onset TauP301S mice and potential mechanisms of action in vivo.

To elicit antibodies that recognize multiple phosphorylation sites while avoiding tau-specific T-cell activation, we designed three synthetic pTau peptides with a combination of up to four AD-related epitopes. pTau30 is phosphorylated at residues Ser202/Thr205/Ser238/Ser262; pTau31 is phosphorylated at residues Ser202/Thr205/Ser396/Ser404; pTau35 is phosphorylated at Ser238/Ser262/Ser396/Ser404 (Fig. 1a, Supplementary Table S1). Immunization tests in C57BL/6 mice (Supplementary Fig. S1a) reflected that only pTau31-induced antibodies could recognize all carried four epitopes (Figs. 1b–d, S1b–f). Furthermore, pTau31 could neither elicit non-phosphorylated Tau31-specific antibodies, nor stimulate tau-specific T-cell activation (Supplementary Fig. S1g, h).

**Fig. 1:**
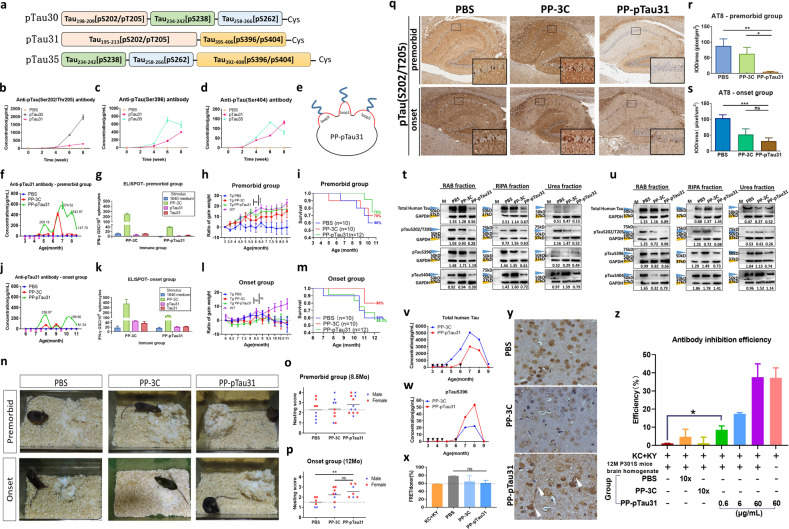
**a** Schematic representation of the three candidate pTau peptides. **b**–**d** Concentrations of pTauS205/T205-, pTauS396-, and pTauS404-specific antibodies induced by the candidate peptide after four doses of immunization. The concentration was calibrated to the AT8 antibody, PHF13 antibody, and pTauS404 polyclonal antibody in (**b**), (**c**), and (**d**), respectively. **e** Schematic representation of the PP-3C-pTau31 vaccine. All three mutant cysteines on the loop of the PP-3C protein (red lines) have an opportunity to bind with the pTau31 peptide (blue lines) during air oxidation in (NH_4_)_2_CO_3_ buffer. **f**, **j** Changes in the concentrations of pTau31-specific serum antibody in TauP301S mice from the premorbid and onset groups. **g**, **k** Levels of T-cell immunoreaction of isolated spleen cells from PP-3C and PP-pTau31 group when stimulated with different stimuli assessed by ELISpot assay in the premorbid and onset groups. All data represent the mean ± SEM. **h**, **l** Ratio of gained weight in TauP301S mice from the premorbid and onset groups during the observation. Weight data of age- and sex-matched wild-type (WT) littermates during the same period are presented as a reference for normal mice. **i**, **m** Survival of TauP301S mice in the premorbid and onset groups during the observation. **n** Representative figures of nest-building behavior for each group. **o**, **p** Nest building test scores of TauP301S mice in the premorbid and onset groups. **q** IHC staining of pTauS202/T205 in the hippocampus of TauP301S mice brain after administration of vaccines. **r**, **s** Quantification of pTauS202/T205 signal stained by AT8 antibody in the hippocampus of TauP301S mice in the premorbid and onset cohorts. The results are expressed as IOD/area. In the premorbid cohort, the PP-pTau31 group showed 93.81% and 91.31% decreases compared to the PBS (*p* = 0.0029) and PP-3C group (*p* = 0.0202), respectively. In the onset cohort, the PP-pTau31 group showed 69.88% and 40.24% decreases compared to the PBS (*p* = 0.0007) and PP-3C groups, respectively. **t**, **u** Levels of human Tau (stained with HT7 antibody), pTauS202/T205 (stained with AT8 antibody), pTauS396 (stained with PHF13 antibody), and pTauS404 in the brain homogenates of mice from the premorbid and onset cohorts after vaccination assessed by western blot assay. GAPDH served as the internal control. The orange arrowheads indicated the Tau or pTau band. The blue arrowheads indicated the GAPDH band. The relative content of each sample was marked under ladders. **v** Concentration of the total human Tau protein in the serum of TauP301S mice in the premorbid cohort during the observation. The black arrowheads indicated the time points of immunization. **w** Level of the pTau protein containing the phosphorylation on Ser396 in the serum of TauP301S mice in the premorbid cohort during the observation. The black arrowheads indicate the time points of immunization. **x** Efficiency of brain homogenate-induced FRET of ΔK280-CFP (KC) and ΔK280-YFP (KY) co-transfected 293T cells of the premorbid cohort. KC + KY represent the baseline, and brain homogenate sample of PBS, PP-3-C, or PP-pTau group was added to the cell medium to test the propagation inhibition activity. **y** Cortex of 9-month-old male TauP301S mice after staining with purified polyclonal antibodies from the immunosera of all mice in the premorbid cohort. Antibodies were diluted 50 folds for detection. Scale bar: 50 μm. **z** The inhibition efficiency of serum antibodies from all mice in the premorbid cohort in inhibiting the toxicity of Tau protein in TauP301S mice homogenate. 293T cells co-transfected with ΔK280-CFP (KC) and ΔK280-YFP (KY) gene served as a biosensor. The RAB fraction of 12-month-old male TauP301S mouse brain homogenate with a concentration of 3 mg/mL total protein was used to induce ΔK280 protein aggregation and toxicity to the cells. Polyclonal antibodies in the serum of immunized mice were purified using ammonium sulfate precipitation, and the terminal volume was concentrated 10 times compared with the input volume. The concentration of polyclonal antibodies in PP-3C-pTau31 serum was calibrated to AT8 antibody by ELISA, and the concentration was 600 μg/mL. Antibodies from the mice in PBS, PP-3C, and PP-pTau31 groups were diluted 10-folds for detection, and the PP-pTau31 vaccine-induced antibodies were further diluted to 6 and 0.6 μg/mL for detection. All data represent the mean ± SEM. *p < 0.05; **p < 0.01; ***p < 0.001; ****p < 0.0001

To increase the immunogenicity and prolong the duration of antibodies, pTau31 peptide was further loaded to PP vaccine carrier and formulated with appropriate adjuvant. NoV P protein was mutated to PP-3C, which was ~34 kD and formed a 20 nm multimer (Supplementary Fig. S2a–c). Then we conjugated pTau31 to the cysteine of PP-3C to form PP-pTau31 vaccine and the conjugation efficiency was 25–40% (Figs. 1e, Supplementary Fig. S2d–g). We have screened the optimal dose of 25 μg/dose for PP-pTau31 vaccine, and found that the combination of CpG and AS02 adjuvant induced the highest pTau31-specific antibody titer in WT mice (Supplementary Fig. S3a–c). Further ELISpot analysis revealed that the PP-pTau31 vaccine did not induce pTau31-specific T-cell activation (Supplementary Fig. S3d). Besides, the application of AS02 plus CpG caused the strongest activation of PP-specific T cells, which provide the additional T cell-mediated help required by anti-pTau-specific B cells (Supplementary Fig. S3e, f).

Next, we investigated the immunogenicity of PP-pTau31 in two cohorts of TauP301S mice (Supplementary Fig. S4a, b). One premorbid cohort was immunized before AD onset at 3-month-old and another onset cohort was treated after AD onset at 6-month-old. After the fourth immunization, PP-pTau31 successfully induced a strong and specific antibody response against pTau31 in both cohorts, with titers of 209–257 μg/mL (Fig. 1f, j). The pTau antibody titer was remarkably enhanced to 575 μg/mL after the boost immunization in premorbid cohort, but failed to increase in onset cohort, which might due to the weak immune system and high deposition of neurofibrillary tangles (NFTs) in older mice. Furthermore, PP-pTau31 induced activation of PP-specific T cells, but could not elicit Tau-specific T-cell activation (Fig. 1g, k). There were no significant changes in serum inflammatory cytokines and chemokines in PP-pTau31-immunized mice compared to PBS controls in both cohorts (Supplementary Fig. S5a, b). PP-pTau31 vaccination also significantly attenuated the activation of microglia and astrocytes in the hippocampus of TauP301S mice (Supplementary Fig. S6a–f).

To determine the functional benefit after PP-pTau31 vaccination, we monitored behavioral changes of TauP301S mice in both cohorts. In premorbid cohort, the weight growth curve of PP-pTau31 vaccinated mice increased fast, and approximately to that of age-matched WT mice, but the weight of TauP301S mice in onset cohort did not change, demonstrating that PP-pTau31 effectively protected AD mice from weight loss (Fig. 1h, l). Furthermore, PP-pTau31 immunization before onset noticeably prolonged the survival time by 15% as compared with PBS treatment, while no survival improvement was found in onset cohort (Fig. 1i, m). In addition, mice immunized with vaccine built better nests in both cohorts, indicating the cognitive improvement by PP-pTau31 (Fig. 1n–p). Moreover, PP-pTau31-immunized mice showed a sustained prolonged latency in the accelerating rotarod test and a slight increase in grip strength in premorbid cohort (Supplementary Fig. S4c–f). TauP301S mice immunized before onset also recovered from muscular atrophy and performed better than control mice in the hind-limb clasping and kyphosis tests (Supplementary Fig. S4i–l).

Then we compared NFT deposition in the hippocampus between PP-pTau31-vaccinated and control mice. PP-pTau31 significantly reduced pTauS202/T205-positive NFTs when compared with PBS in both cohorts, and the relative area covered by pTauS396- and pTauS404- (Figs. 1q–s, Supplementary Fig. S6g–l) positive NFTs in PP-pTau31-vaccinated mice was also reduced obviously. We also measured total Tau and pTau levels in brain homogenates. In premorbid cohort, total Tau levels in the soluble fractions of brain homogenates decreased sharply after vaccination (Fig. 1t). In addition, PP-pTau31 treatment before onset markedly reduced the levels of pTauS202/T205, pTauS396, and pTauS404 in all three brain fractions (Fig. 1t). In onset cohort, the reduction of pTau protein was only observed in the RIPA and Urea fraction following vaccination (Fig. 1u).

We further explored the potential mechanism of PP-pTau31. The serum total human tau in PP-pTau31-immunized TauP301S mice was obviously lower than controls, especially between 6- and 8-month of age, which coincided with a sharp increase in serum pTau antibodies during the same time (Fig. 1v). Oppositely, the serum concentration of pTauS396 in PP-pTau31-treated mice increased faster than control at this period (Fig. 1w), suggesting that PP-pTau31-elicited antibodies accelerated the efflux of pTau protein from the brain by combining with the pTau protein in the peripheral circulation system. Pathological tau spreads extracellularly and induces misfolding of intracellular tau.^4,5^ Therefore, we investigated the propagation of pathological tau in mice brain homogenates using FRET. We found that the FRET signal in the PP-pTau31 group showed a moderate decrease compared with PBS controls (Fig. 1x). Purified PP-pTau31-elicited antibodies, which specifically recognized NFTs in TauP301S mice, could also significantly enhanced inhibition of pathological tau-induced toxicity in a dose-dependent manner (Fig. 1y, z).

In summary, PP-pTau31-generated robust pTau antibodies could significantly reduce tau pathology and improve behavioral deficits. PP-pTau31 might represent a novel promising immunotherapeutic strategy against tau pathology in AD.

## Supplementary information

Supplementary Materials
