# Correction to: Norovirus P particle-based tau vaccine-generated phosphorylated tau antibodies markedly ameliorate tau pathology and improve behavioral deficits in mouse model of Alzheimer’s disease

**DOI:** 10.1038/s41392-021-00657-6

**Published:** 2021-06-14

**Authors:** Yao Sun, Yongqing Guo, Xuejian Feng, Lu Fu, Yayuan Zheng, Yue Dong, Yong Zhang, Xianghui Yu, Wei Kong, Hui Wu

**Affiliations:** 1 National Engineering Laboratory for AIDS Vaccine, School of Life Sciences, Jilin University, Changchun, 130012 China; 2 Laboratory of Pathogenic Microbiology and Immunology, College of Life Science, Jilin Agricultural University, Changchun, 130012 China; 3 Key Laboratory for Molecular Enzymology and Engineering, the Ministry of Education, School of Life Sciences, Jilin University, Changchun, 130012 China

**Keywords:** Vaccines, Diseases of the nervous system

Correction to: *Signal Transduction and Targeted Therapy* 10.1038/s41392-020-00416-z, published online 13 February 2021

In the process of collating the published data, the authors noticed one inadvertent mistake occurred during the production process in Fig. 1u that needs to be corrected.^1^ The authors mistakenly placed the wrong western blot figure for the level of pTauS404 in the urea fraction of mice from the onset cohort in Fig. 1u. The correct data are provided as follows. The key findings of the article are not affected by these corrections. The original article has been corrected.

**Fig. 1:**
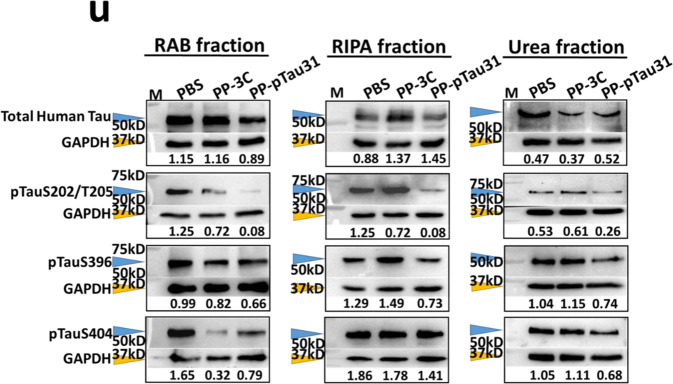
(**u**) Levels of human Tau (stained with HT7 antibody), pTauS202/T205 (stained with AT8 antibody), pTauS396 (stained with PHF13 antibody), and pTauS404 in the brain homogenates of mice from the onset cohorts after vaccination assessed by western blot assay. GAPDH served as the internal control. The orange arrowheads indicated the Tau or pTau band. The blue arrowheads indicated the GAPDH band. The relative content of each sample was marked under ladders

## References

[1] Sun Y (2021). Norovirus P particle-based tau vaccine-generated phosphorylated tau antibodies markedly ameliorate tau pathology and improve behavioral deficits in mouse model of Alzheimer’s disease. Signal Transduct. Target. Ther..

