# Supplementary material for: Norovirus P particle-based tau vaccine-generated phosphorylated tau antibodies markedly ameliorate tau pathology and improve behavioral deficits in mouse model of Alzheimer’s disease
Source: Signal Transduct Target Ther. 2021 Feb 13;6:61. doi: 10.1038/s41392-020-00416-z (PMC7881025; doi:10.1038/s41392-020-00416-z)
Supplement: Supplementary file 1 — Supplementary Materials [file 41392_2020_416_MOESM1_ESM.docx]

Supplementary Materials for

**Norovirus P particle-based tau vaccine-generated phosphorylated tau antibodies** **markedly ameliorate tau pathology and improve behavioral deficits in mouse model of Alzheimer’s disease**

Yao Sun, Yongqing Guo, Xuejian Feng, Lu Fu, Yayuan Zheng, Yue Dong,

Yong Zhang, Xianghui Yu, Wei Kong, Hui Wu

Correspondence to: [topwuhui@jlu.edu.cn](mailto:topwuhui@jlu.edu.cn)

**This PDF file includes:**

Materials and Methods

References for “Material and Methods” section

Figures. S1 to S10

Table S1-S2

**Materials and methods**

**Expression constructs**

The NoV P domain (Hu/GII.4 GenBank: AAZ31396.2) in a pET28a(+) vector was used as a template for inducing a cysteine point mutation in each of the three surface loops (primers sequence (5’→3’):

T73C-F: CTGTGAACATCGCTACTTTCCGCGGCGACGTCACACACATCGCTTGCACACAAAACTAC;

T73C-R: GTAGTTTTGTGTGCAAGCGATGTGTGTGACGTCGCCGCGGAAAGTAGCGATGTTCACAG;

S149C-F: GCAATTCAGCACAGACACCTGCAACGATTTCGAGACTGGCC; S149C-R: GGCCAGTCTCGAAATCGTTGCAGGTGTCTGTGCTGAATTGC; S169C-F: CCGTGGGTGTCGTTCAAGACTGCAGCACCACTCACCAGAACG; S169C-R: CGTTCTGGTGAGTGGTGCTGCAGTCTTGAACGACACCCACGG), and adding Hig tag in the C terminus of protein, and then transferred into the pCold IV vector (cat. No. 3364, Takara, Japan) to produce a recombinant protein, PP-3C. The human tau (0N4R) sequence in pCR-BluntⅡ-TOPO vector was purchased from GE Life Sciences (clone ID: 40007445, cat. No. MHS6278-211689579) and was cloned into a pET28a(+) vector for protein expression.

**Protein expression and purification**

The PP-3C and tau proteins were expressed in *Escherichia coli* Chaperone Competent Cells PGro7/BL21 (cat. No. 9122, Takara. Japan) and *E. coli* strain BL21 (DE3) Chemically Competent Cells (cat. No. CD601-01, TransGen Biotech, Beijing, China), respectively. PP-3C was purified by Ni-NTA affinity chromatography, as reported previously.^1^ The protein fraction eluted with 300 mM imidazole was used for further analysis. PP-3C protein buffer was replaced with 100 mM ammonium bicarbonate (pH, 8.0) using desalting chromatography (HiPrep 26/10 Desalting; GE Healthcare Life Sciences) for subsequent binding tests with synthetic peptides.

**Sodium dodecyl sulfate (SDS)-polyacrylamide gel electrophoresis (PAGE), native PAGE, and transmission electron microscopy (TEM)**

The molecular weights of the proteins were estimated by 13.5% SDS-PAGE, followed by Coomassie blue staining. The 24-mer form of PP-3C protein was identified using native PAGE, followed by Coomassie blue staining. No SDS, β-mercaptoethanol, or dithiothreitol was used in the gel-electrophoresis system. The morphological characteristics of the proteins were observed by TEM (EM-11210SQCH; JEOL, Japan), using an accelerating voltage of 80 kV, and images (50 k× magnification) were obtained with a CCD camera system.

**Synthetic tau peptides**

All peptides were synthesized at GL Biochem (Shanghai, China). The peptide sequences are listed in Table S1. A cysteine was added to the C terminus of the peptides for conjugation to the PP-3C protein. Bovine serum albumin (BSA)-conjugated peptides were used as antigens in the coating step in ELISA, as this conjugate produced the lowest background in preliminary experiments (data not shown).

**pTau31 peptide conjugation to PP-3C protein**

The pTau31 peptide was diluted in a NH_4_HCO_3_ solution (pH, 8.0) containing PP-3C protein at a molar ratio of 30:1 and continuously rotated at 4°C for 60 h. Then, the mixture was transferred into a centrifugal filter (Amicon® Ultra-4 Centrifugal Filter Unit, 10-kD cut-off; Millipore) and centrifuged at 3000 ×*g* for 10 min followed by resuspension in 4 mL of phosphate-buffered saline (PBS); centrifugation was repeated four times to remove the free peptide. The PP-pTau31 concentration was 0.5 mg/mL. The conjugate was mixed with dithiothreitol (DTT) at a final concentration of 2 mM and left to stand at 20–25°C for 16 h to disaggregate pTau31 from PP-3C. The reaction product was centrifuged at 20000 ×*g* for 5 min, and the supernatant was collected to detect the free pTau31 by high-performance liquid chromatography (HPLC). The PP-3C concentration was determined by a BSA quantification assay. The conjugation ratio of PP-pTau31 was calculated as follows:

$$conjugation ratio=\frac{concentration of pTau31\times Mw(PP-3C)}{3\times concentration of (PP-3C)\times Mw(pTau31)}=\frac{35.86\times concentration of pTau31}{3\times3.15\times concentration of (PP-3C)}$$

**HPLC**

The amount of free peptides in the buffer was determined by HPLC. Standard pTau31 was diluted in PBS to a final concentration of 1 mg/mL, and 2-fold serial peptide dilutions were detected using reversed-phase C18 columns (Agilent Technologies, SB-C18, 4.6×250 mm USA) under a continuous gradient mixture of water and acetonitrile. A pTau31 standard curve was used to calculate the concentration of free peptide in the samples.

**Animal experiments and ethics committee approval**

All animals were housed in individually ventilated cages (n=2/3 per cage) at the Animal Experimental Platform, Core Facilities for Life Science, Jilin University, at 20–22°C and 40–60% humidity under a 12-h dark/light cycle. All experiments were performed in accordance with legal and institutional guidelines and were approved by the Ethical Committee of Care and Use of Laboratory Animals at Jilin University.

**Immunogenicity of the peptide in C57BL/6 mice**

Forty-eight 8-week-old female C57BL/6 mice were used to determine the peptide with the highest immunogenicity while inducing a low T-cell immune response. pTau30, pTau31, and pTau35 peptides (Table S1) were diluted at 2 mg/mL in PBS and then mixed with complete Freund’s adjuvant (used in the first dose of immunization) or incomplete Freund’s adjuvant (used in the second to fourth doses of immunization, both adjuvants were from Beijing Dingguo Chang Sheng Biotechnology, China) at a ratio of 1:1 (v/v). One milliliter of the mixture was subjected to ultrasound irradiation under ice-water immersion at a power of 30% for a total of 60 s at 5 s intervals to form a stable emulsion. Three groups of six mice were administrated 100 μL of peptide vaccine emulsion in both hind legs via intramuscular injection every two weeks, and PBS was used as a blank control. Blood samples were collected from the submandibular vein plexus before immunization, incubated at 37°C for 2 h, and centrifuged at 1150 ×*g* at 4°C for 30 min. The serum was transferred into EP tubes, and stored at –20°C, avoiding freeze/thaw. The serum was used for ELISA to detect specific antibodies induced by immunization. The mice were euthanized using CO_2_ three weeks after the last dose of immunization, and the spleen was aseptically removed for enzyme-linked immune absorbent spot (ELISpot) assays.

**Dose-response and optimal PP-pTau31 adjuvant studies in C57BL/6 mice**

Forty-eight 8-week-old female C57BL/6 mice were used to determine the optimal immunogenic dose of and optimal adjuvant for the PP-3C-pTau31 vaccine. The mice were randomly divided into eight groups of six mice. One lot of 0.42 mg/mL PP-pTau31 with an average pTau31–PP-3C conjugation ratio of 25% was used as the antigen and diluted with sterile PBS before immunization. Three groups of mice were immunized with gradient doses of PP-3C-pTau31 (12.5 μg/100 μL/dose, 25 μg/100 μL/dose, and 50 μg/120 μL/dose). Another four groups of mice received 25 μg PP-pTau31 combined with cytosine-phosphoguanine (CpG), AS02+CpG, AS03+CpG, or MF59+CpG adjuvant. CpG-enriched oligodeoxynucleotide (5′-TGTCGTCGTCGTTTGTCGTTTGTCGTT-3′, Takara (Dalian) Bioengineering) was diluted in PBS and added to the vaccine at a final concentration of 10 ng/dose. The preparation of the other adjuvants has been described in previous reports.^2,3^ In brief, MF59 comprises 5.0% (v/v) squalene, 0.5% (v/v) polysorbate-80, and 0.5% (v/v) sorbitane trioleate in sodium citrate buffer (pH 6.5); AS03 comprises 5.0% (v/v) squalene, 1.8% (v/v) polysorbate-80, and 5% (v/v) dl-α-tocopherol in PBS (pH, 6.8); and AS02 is a combination of AS03, MPLA, and saponin QS21 (*Quillaja saponaria* Molina, fraction 21). MF59, AS02, or AS03 was added into the mixture of antigen solution and CpG solution at a ratio of 1:1 (v/v). PBS was used as a blank control. Each group was immunized at time zero and week two, four, six, and twelve via intramuscular injection. Serum was collected before immunization for ELISA, and the spleen was collected for ELISpot assays.

**P301S transgenic mice and vaccine immunization**

Heterozygous transgenic mice (Tg(Prnp-MAPT*P301s)Ps19Vle/JNju) on B6C3 background (P301S mice) were purchased from the Model Animal Research Center of Nanjing University (Nanjing, China). These mice express the P301S human 1N4R tau isoform under the murine prion protein (Prnp) promoter. Genetic background- and age-matched non-transgenic littermates were used as wild type (WT) control. Three- and six-month-old mice were assigned to a premorbid treatment cohort and an onset treatment cohort, respectively, to test the effectiveness of the vaccine. Three groups of P301S mice, which consist males and females, were assigned the following treatments, administered via intramuscular injection: 100 μL PBS (n=10), 100 μL PP-3C with AS02+CpG adjuvant (25 μg/dose in the premorbid cohort and 50 μg/dose in the onset cohort, n=10), and 100 μL PP-pTau31 vaccine with AS02+CpG adjuvant (25 μg/dose in the premorbid cohort and 50 μg/dose in the onset cohort, n=12). A group of 10 female WT mice was administered PBS for monitoring body weight changes throughout the experimental period. Each group of mice was administered four doses at two-week intervals, followed by a booster dose at the fourth or sixth week after the fourth dose immunization in the premorbid and onset cohorts, respectively. Blood samples were collected at two-week intervals. Serum was used to measure the concentrations of specific antibody, total human tau, pTau (Ser396), chemokines, and inflammatory cytokines.

During immunotherapy of the AD mice, we continuously monitored behavioral changes by measuring body weight and using accelerating rotarod, grip strength once a month. Composite phenotype scoring system, and nest building tests were performed after bleeding progress (Fig. 4A-B). The methods of these behavioral tests were described by Sun *et al*..^4^ At the end of experiments, the mice were deeply anesthetized with pentobarbital by intraperitoneal injection (100 mg/kg), perfused with PBS, and organs were harvested for pathological examination.

**Pathologic examination of the brain and other organs**

The mouse brains were separated into two hemispheres after removal. One hemisphere was deep-frozen in liquid nitrogen and homogenized, and further divided into reassembly buffer (RAB) fraction, radio immunoprecipitation assay (RIPA) fraction and Urea fraction as described before.^4^ The brain homogenate fraction samples of each mice from the same group were mixed in a ratio of 1:1 before western blot analysis, which represent the average total Tau protein of pTau protein levels of the corresponding group. The other hemisphere was immersed in 4% paraformaldehyde for immunohistochemistry (IHC) to detect the levels of NFT, microglia and astrocytes. Heart, liver, spleen, and kidneys were collected and immersed in 4% paraformaldehyde for hematoxylin and eosin (H&E) staining. The brain homogenates were used for western blot analysis, fluorescence resonance energy transfer (FRET), and the detection of inflammatory factors and chemokines by flow cytometry as described by Sun *et al*..^4^

**Western blot (WB)**

Protein samples were separated by 13.5% SDS-PAGE, then transferred onto membranes for immunoblot assay. 0.45 μm nitrocellulose membranes (Whatman, Kent, UK) was used for RAB or RIPA fraction of brain homogenate and purified protein samples, and 0.22 μm PVDF membranes (GE healthcare, USA) was used for Urea fraction of brain homogenate. Briefly, blotting membranes incubated with 10% skimmed milk for 20 min at room temperature, then incubated with primary antibodies at 2-8°C overnight, followed by react with horseradish peroxidase (HRP)- conjugated secondary antibody for 1 h at room temperature. After wash membranes with PBS, they were incubated with ECL solution, and exposed under a chemiluminescent imaging system (Tanon 6100, Tanon Science & Technology Co,. Ltd., Shanghai, China). Antibodies and reagents information were listed in Table S2. The relative quantification of target protein to GAPDH of all ladders were analyzed by Image J (National Institutes of Health, USA).

**Dot blot**

0.45 μm nitrocellulose membranes were activated with transfer buffer (47 mM Tris, 50 mM glycine, 20% methanol (v/v)) for 5 min, then transferred into Bio-Dot® Microfiltration apparatus (cat. No. 1073938, Bio-Rad Laboratories, Inc. USA). Add diluted protein samples onto membranes, then incubated them with 10% skimmed milk, antibodies and ECL solution as describe in Western blot section.

**Cytokine (T-cell activation) determination by ELISpot assay**

The Mouse IFN-γ ELISpot Kit (BD Biosciences, USA) was used to analyze T-cell activation in each group. The spleens of the mice were removed aseptically and single-cell suspensions were prepared. After cell counting, cells were seeded at 1 × 10^6^ cells/well in 100 μL of 1640 medium into 96-well plates coated with anti-IFN-γ antibody in triplicate. The cells were stimulated with 100 ng/mL peptide (pTau31, Tau31, pTau202/205, pTau396 or pTau404) or protein (tau protein or PP-3C protein), or with Concanavalin A (cat. No. S12028, Shanghai yuanye Bio-Technology, Shanghai, China) as a positive control. The plates were incubated at 37°C in the presence of 5% CO_2_ for 24 h. Positive spots were counted according to the manufacturer’s recommendations.

**Purification of serum antibody from immunized mice**

Polyclonal antibodies in antisera of the immunized mice were purified using saturated ammonium sulfate (SAS) precipitation. Five hundred microliters of antiserum were diluted with 0.5 mL of 0.9% NaCl, and 1 mL of SAS was slowly dropped into the dilution, which was incubated statically for 20 min. The mixture was centrifuged at 10000 ×*g* for 20 min and the pellet was resuspended in 2 mL of 0.9% NaCl. Then, 0.5 mL of SAS was slowly dropped into the supernatant, which was incubated statically for 20 min. The mixture was centrifuged again, and the pellet was resuspended in 0.5 mL of 0.9% NaCl. During the purification process, the samples were kept at 2–8°C. The purified antibody was quantified using ELISA.

**Fluorescence resonance energy transfer (FRET)**

The toxicity and propagability of soluble tau protein in mice homogenates were confirmed in a cell model by FRET as described previously,^5^ with modification to allow detection using a fluorescence spectrometer (LS55; PerkinElmer, USA).

A truncated human tau sequence encoding amino acids 243–375 (repeat domain, RD) was cloned into pcDNA3.1(+). P301L&V337M (LM) (internal positive control) and △K280 (△K) mutations were introduced for experiments. In addition, we added cyan fluorescent protein (CFP) or yellow fluorescent protein (YFP) to the C terminus of △K to generate △K-CFP (KC) and △K-YFP (KY), respectively.

293T cells were cultured in Dulbecco’s Modified Eagle’s Medium (DMEM) supplemented with 10% fetal bovine serum. For transient transfection, 293T cells were harvested with 0.10% trypsin and transferred to a 6-well plate (1.2 × 10^6^ cells/well) for 24 h. Subsequently, Lipofectamine^TM^ 2000 transfection reagent (cat. No. 11668019, ThermoFisherScientific, USA) and 2.4 μg of LM or △K-YFP plasmid diluted in 1 mL of DMEM with antibiotics (100 μg/mL penicillin, 100 μg/mL streptomycin) were added to each well. Co-transfected cells received a combination of 0.6 μg △K-CFP and 1.8 μg △K-YFP per well. After 16 h, the cells were harvested and transferred to 12-well plates (2 × 10^5^ cells/well) in the presence or absence of 10 μL of the RAB fraction of the brain homogenates. To detect the inhibition efficiency of the antibody, diluted antibody solution was mixed with 10 μL of brain homogenate at 4°C for 24 h before mixing with the cells. The cells were harvested into a single EP tube and washed once with sterile PBS. Then, the cells were resuspended in 0.5 mL of sterile PBS, and fluorescence excitation spectra were measured at 435 nm or 485nm, using an emission wavelength of 485 nm or 528nm. Each sample was detected in triplicate, and the FRET/donor value was calculated as follows ^5^:

$${FRET}_{Smpl}=\frac{{Smpl}_{435ex/485em}- \frac{{\triangle K-YFP}_{435ex/528em}}{{\triangle K-YFP}_{485ex/528em}}\times{Smpl}_{485ex/528em}}{{Smpl}_{435ex/485em}}$$

**Statistical analysis**

Statistical analysis was carried out using SPSS software (version 20.0, IL, Chicago, USA) and GraphPad Prism 8.0 (GraphPad Software), with a confidence level set at 95%. Data are presented as the mean ± standard error of the mean (SEM). Relationships between measured behavioral and pathological data collected at consecutive time points were evaluated by Mauchly’s test of sphericity, and multivariate ANOVA was employed when *p* was ≤ 0.05, and the Bonferroni-corrected *t*-test was applied otherwise.

**Data availability**

The datasets analyzed during the current study are available from the corresponding author upon reasonable request.

**References**

1. Fu, L. *et al.* Norovirus P particle: An excellent vaccine platform for antibody production against Alzheimer’s disease. *Immunol. Lett.* **168**, 22–30 (2015).
2. Shi, W. et al. Comparison of immunogenicity, efficacy and transcriptome changes of inactivated rabies virus vaccine with different adjuvants. *Vaccine* **36**, 5020–5029 (2018).
3. Ott, G., Radhakrishnan, R., Fang, J.-H. & Hora, M. The Adjuvant MF59: A 10-Year Perspective. in Vaccine Adjuvants 211–228 (Humana Press, 2003). doi:10.1385/1-59259-083-7:211.
4. Sun, Y. *et al.* The behavioural and neuropathologic sexual dimorphism and absence of MIP-3α in tau P301S mouse model of Alzheimer’s disease. *J Neuroinflammation* **17** (2020).
5. Kfoury, N., Holmes, B. B., Jiang, H., Holtzman, D. M. & Diamond, M. I. Trans-cellular Propagation of Tau Aggregation by Fibrillar Species. *J. Biol. Chem.* **287**, 19440–19451 (2012).

**Figure S1**


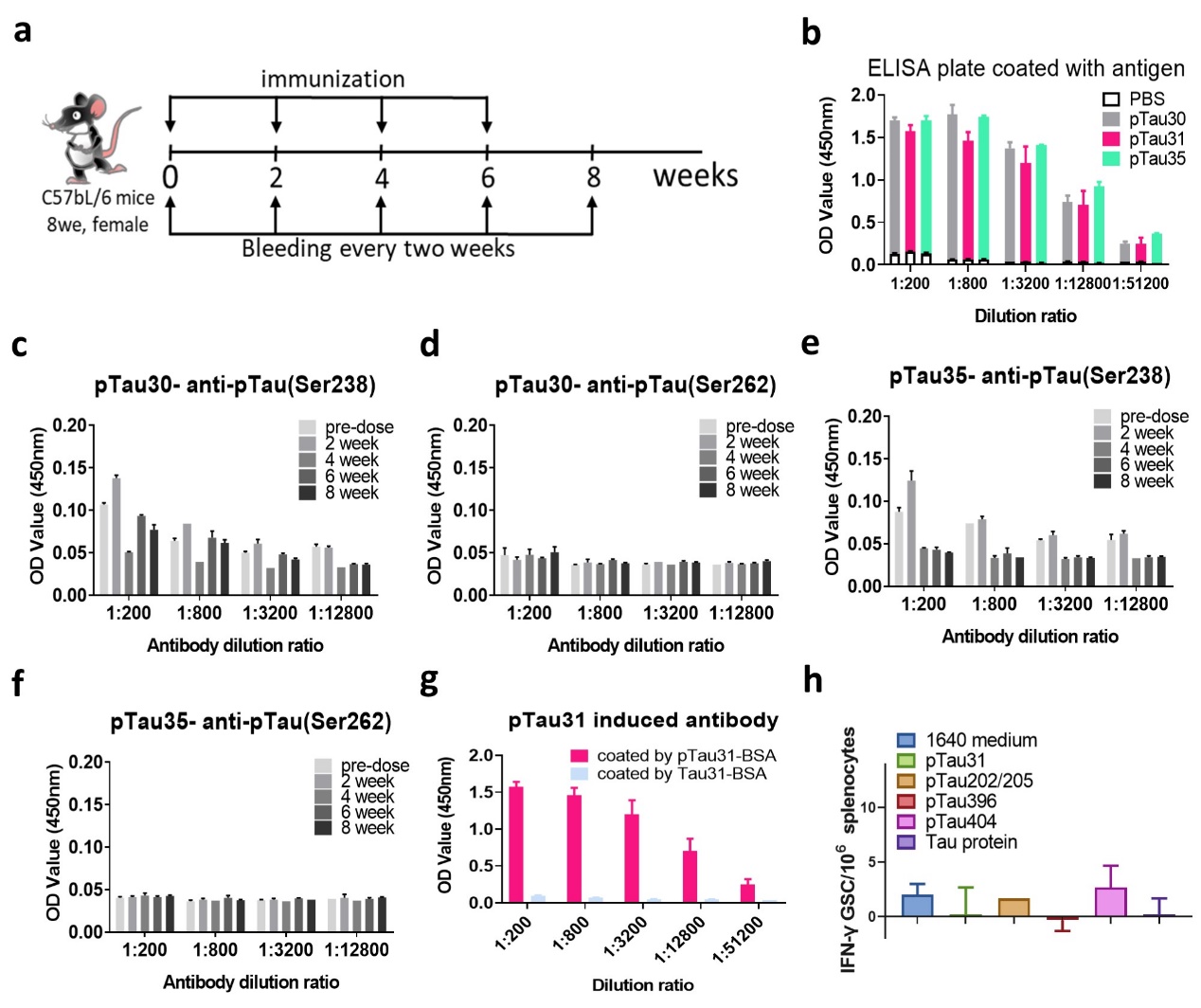


**Fig. S1. Screen of the optimal combination of pTau epitopes.** PBS treatment served as the control. (**a)** Procedure for the immunization and blood collection in the female C57BL/6 mice (n=6/group), all mice were injected with 100μg per dose of peptide vaccine with Freund’s adjuvant (50μL/leg). **(b)** ELISA signals of pTau30-specific (grey), pTau31-specific (pink), and pTau35-specific (green) antibodies in each group after the 4^th^ immunization. The PBS group shows the background values of the three antigen-specific antibodies. (**c,** **d)** ELISA signals of pTauS238- and pTauS262-specific antibodies elicited by pTau30 peptide at each time point. The ELISA plates were coated with pTau238-BSA and pTau262-BSA. (**e,** **f)** ELISA signals of pTauS238- and pTauS262-specific antibodies elicited by pTau35 peptide at each time point. (**g)** ELISA signals of pTau31 peptide-induced phosphorylated Tau31-specific (plate coated with pTau31-BSA) or unphosphorylated Tau31-specific (plate coated with Tau31-BSA) antibodies after the fourth immunization. (**h)** Spleen cells isolated from pTau31 immunization group were stimulated with phosphorylated peptides (pTau31, pTauS202/T205, pTauS396, and pTauS404) and Tau protein, and the IFN-γ secreting T-cell spots were quantified by ELISpot assay. All data represent the mean ± SEM.

**Figure S2**


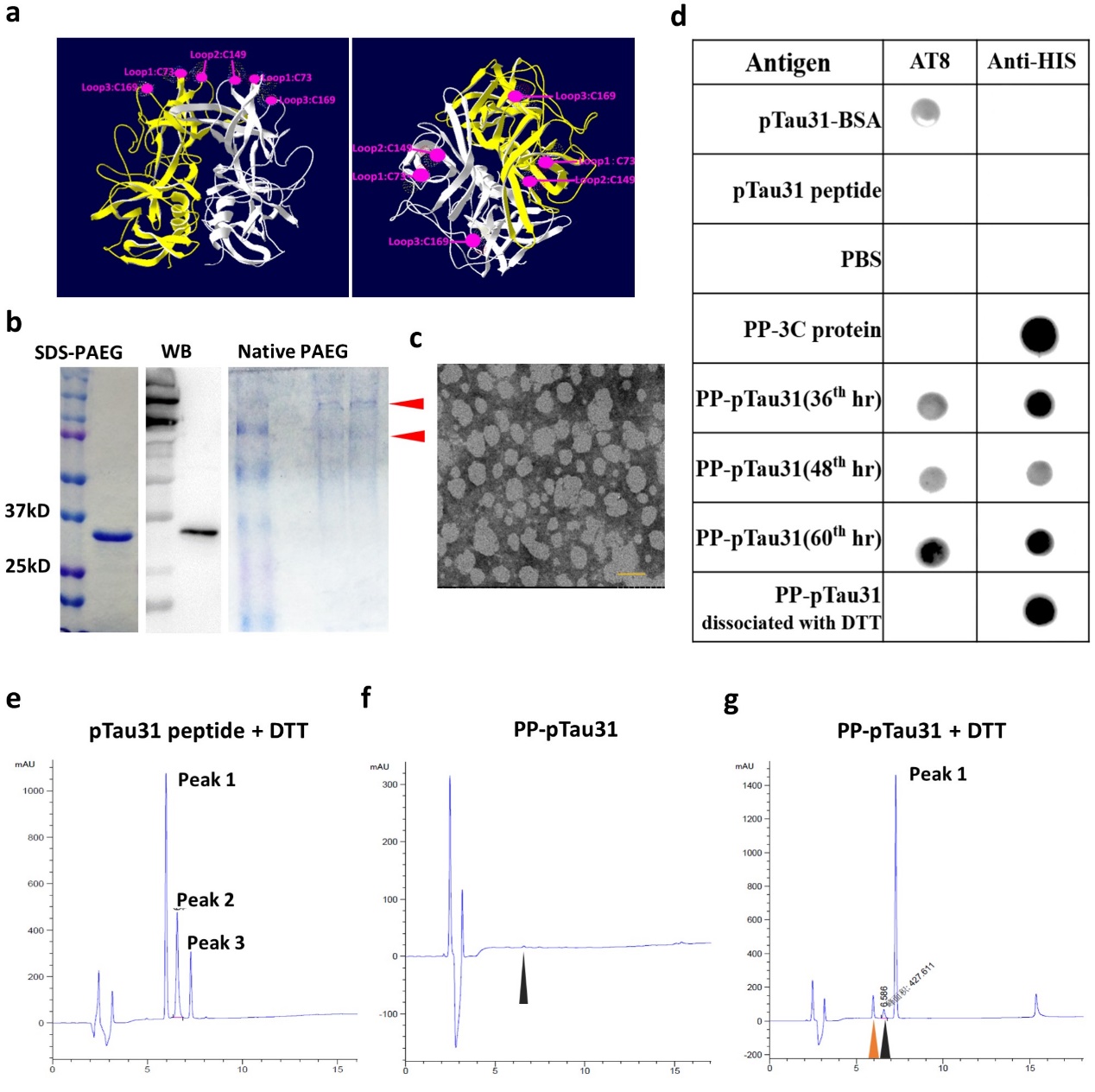


**Fig. S2. Purification of PP-3C protein and preparation of PP-3C-pTau31 vaccine. (a)** Prediction of the homology dimer form of PP-3C protein. The pink sites on the top of backbone represent the cysteine mutation in three loops. (**b)** Recombinant PP-3C protein was analyzed by SDS-PAGE, western blotting (anti-His), and native PAGE. The red arrow indicates the polymer form of PP-3C protein. (**c)** TEM image of PP-3C protein. Scale bar: 50 nm. **(d)** Qualitative analysis of PP-3C-pTau31 by dot blotting. A 10μL sample from each round of PP-3C-pTau31 vaccine preparation was added onto a nitrocellulose membrane, and AT8 antibody (anti-pTauS202/T205) and anti-His antibody was used to identify the pTau31 epitope and PP-3C protein, respectively. Free pTau31 peptide could not be detected by this assay. PTau31 linked to the PP-3C protein was detected after incubation at 4°C for 36 h, and was dissociated with DTT for 12 h. **(e)** Chromatogram of a mixture of pTau31 and DTT by HPLC using a C18 reversed-phase column. Peak 1 corresponds to reduced DTT, Peak 2 corresponds to the pTau31 peptide, Peak 3 corresponds to oxidized DTT. Peak 2 appeared at 6.5 min. **(f)** Chromatogram of the supernatant of denatured PP-pTau31 by HPLC using a C18 reversed-phase column. There is no peak at 6.5 min (black arrow), indicating no free pTau31 in PP-pTau31 production after purification. **(g)** PP-pTau31 was dissociated with DTT overnight, and the supernatant was collected to detect free pTau31 by HPLC using a C18 reversed-phase column. The chromatogram is shown. The yellow arrow indicates reduced DTT, the black arrow indicates dissociated pTau31, Peak 1 corresponds to oxidized DTT.

**Figure S3**


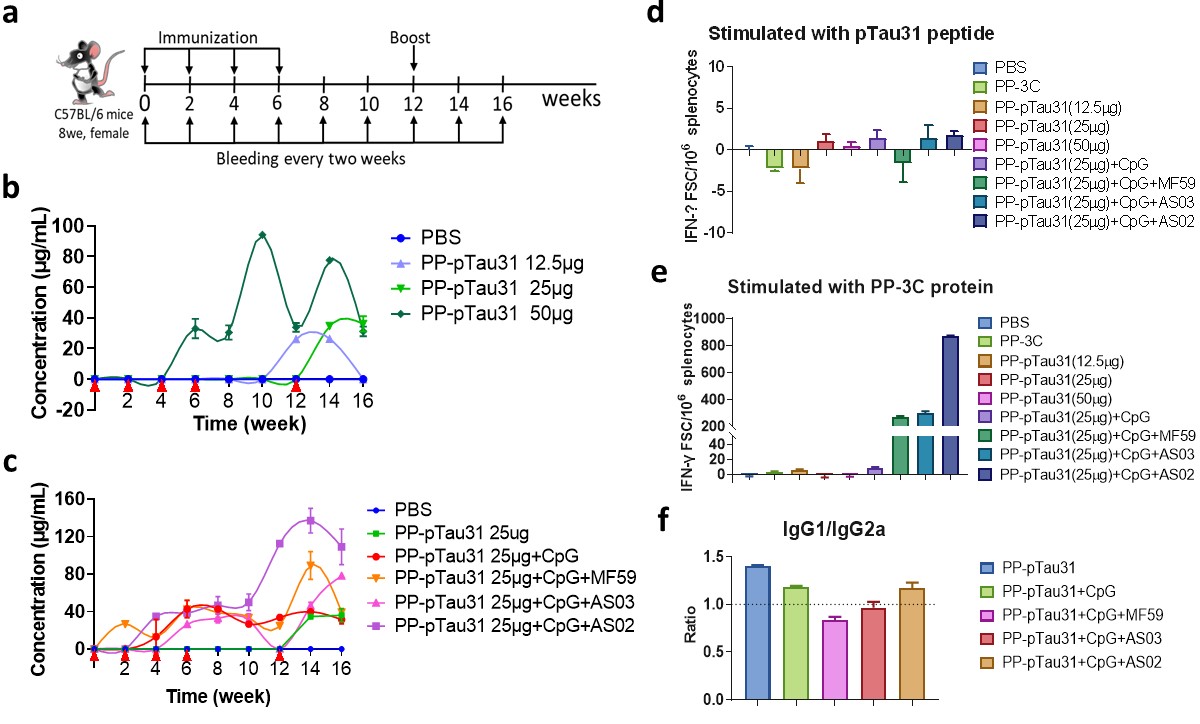


**Fig. S3. Screen for the optimal dose and adjuvants for the PP-pTau31 vaccine in WT mice. (a)** Procedure of immunization and blood collection in female C57BL/6 mice. **(b)** Concentration of pTau31-specific antibody induced by immunization with different dose of PP-pTau31. The red arrowheads indicate the time points of administration. The concentration was calibrated to AT8 antibody. The maximum intramuscular injection volume was 100μL, and the concentration of PP-pTau31 protein was approximately 0.5mg/mL. Considering that addition of the adjuvant at a volume ratio of 1:1 results in a two-fold dilution of the vaccine content, we chose a dose of 25 μg as the optimal dose to suit the volume limitation for administration in mice. **(c)** Concentration of pTau31-specific antibody induced by PP-pTau31 vaccine with different adjuvants during immunization. Red arrowheads indicate the time points of administration. **(d,** **e)** Levels of T-cell immunoreaction of isolated spleen cells from each group when stimulated with pTau31 peptide or PP-3C protein assessed by ELISpot assay. (**f**) ELISA signal ratio of IgG1 to IgG2a of pTau31-specific antibody in each group at the 14^th^ week.

**Figure S4**


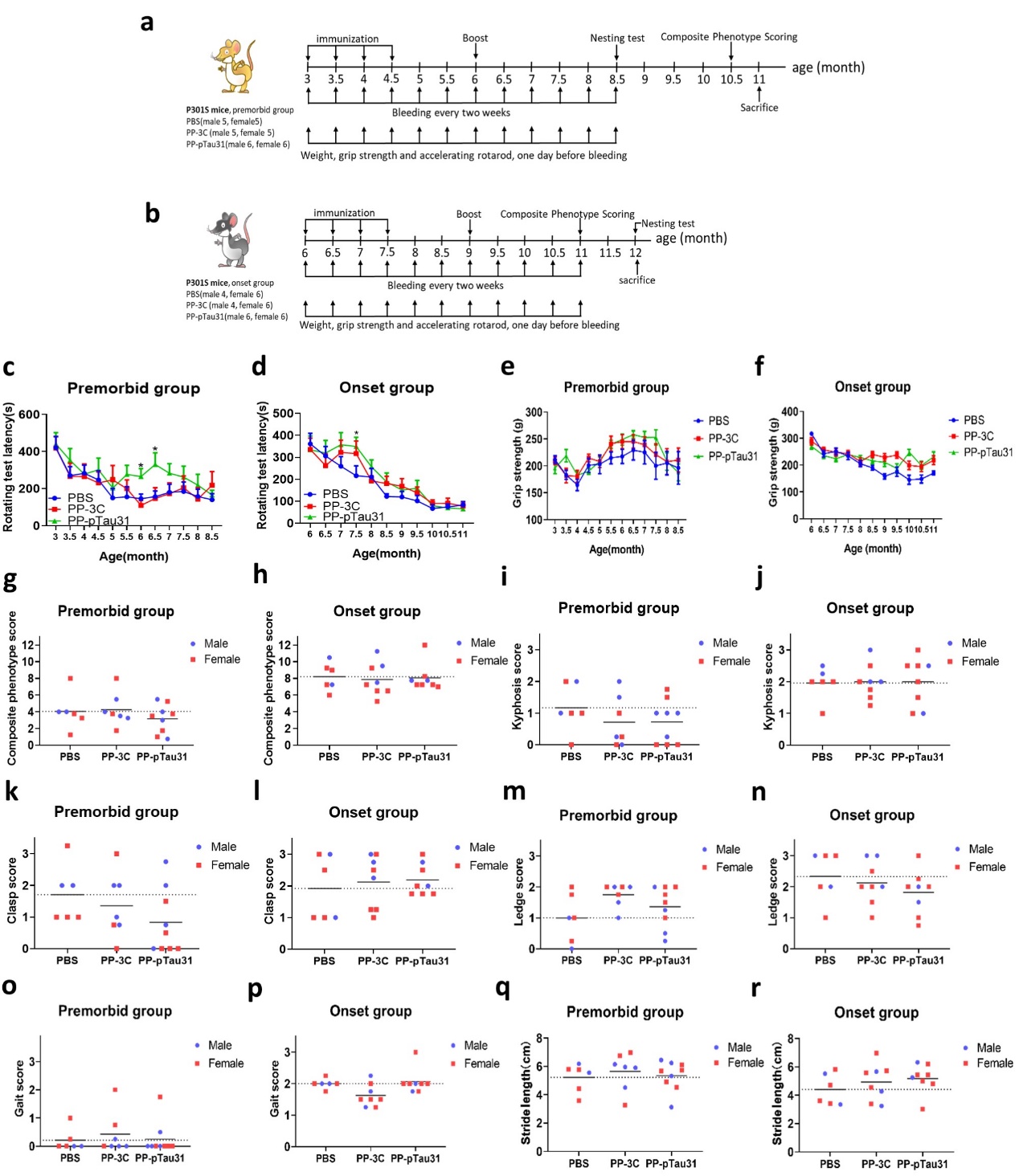


**Fig. S4. The behavioral changes after PP-pTau31 immunization in TauP301S mice from the premorbid and onset cohorts. (a,** **b)** Procedure of immunization, blood collection and behavioral tests in the premorbid group (administration started at 3 months of age) and onset group (administration started at 6 months of age), respectively. Changes in the grip strength in the premorbid cohort (**c**) and onset cohort (**d**) during the observation. (**e, f**) Latency of accelerating rotating test in the premorbid and onset cohorts. **(g**, **h)** Total score of the composite phenotype test (including kyphosis, clasping, ledge and gait) in the premorbid and onset cohorts. **(i,** **j)** Kyphosis test scores in the premorbid and onset cohorts. **(k,** **l)** Clasping test scores in the premorbid cohort (10.5-month-old) and onset cohort (11-month-old). **(m**, **n)** Ledge scores in the premorbid cohort (10.5-month-old) and onset cohort (11-month-old). (**o**, **p)** Gait scores in the premorbid cohort (10.5-month-old) and onset cohort (11-month-old). (**q**, **r)** Stride length of mice in the premorbid cohort (10.5-month-old) and onset cohort (11-month-old).

**Figure S5**

**
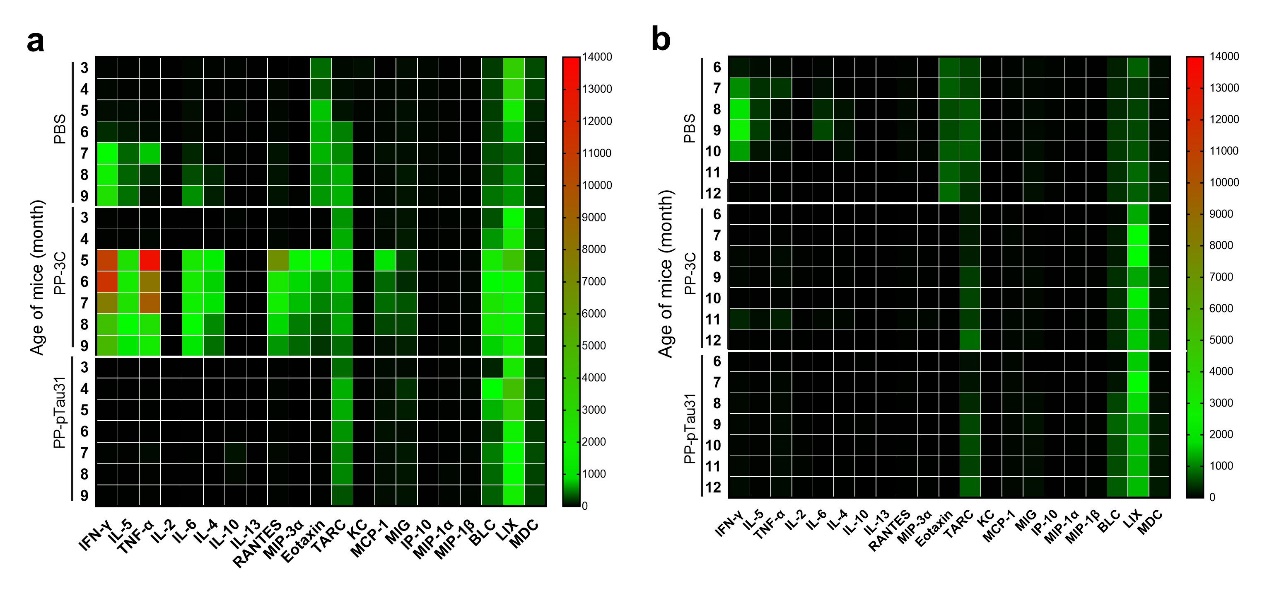
**

**Fig. S5. Changes in the inflammatory cytokines and chemokines in the serum of TauP301S mice in the premorbid cohort (a) and onset cohort (b).**

**Figure S6**


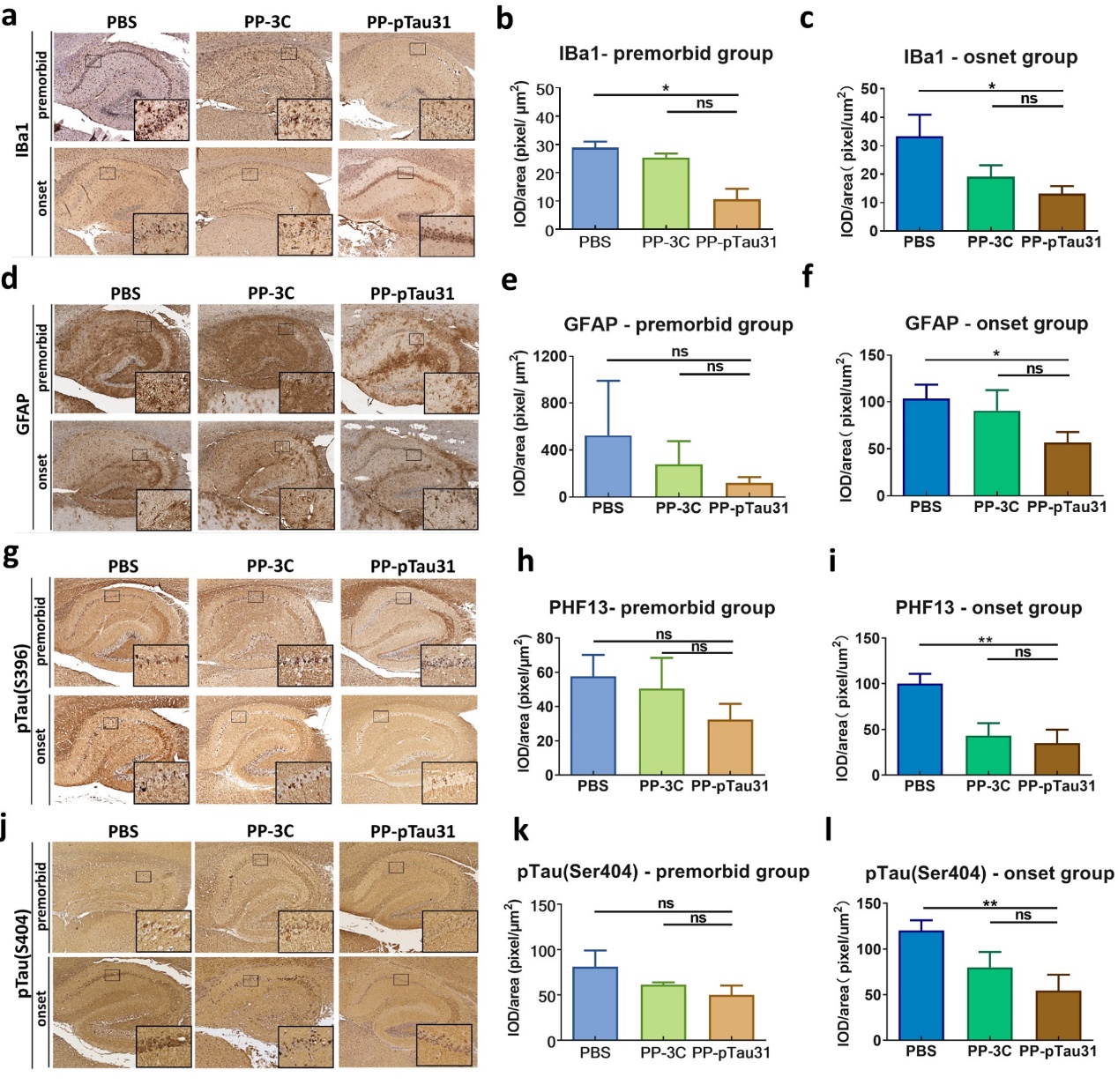


**Fig. S6. Levels of phosphorylated Tau, microglia and astrocytes in the hippocampus slides of TauP301S mice after vaccination. (a)** IHC staining of microglia in the hippocampus of TauP301S mice brain after administration of vaccines. **(b, c)** Quantification of microglia signal stained by IBa1 antibody in the hippocampus of TauP301S mice in the premorbid and onset cohorts. The results are expressed as IOD/area. In the premorbid cohort, the PP-pTau31 group showed 63.30% and 58.21% decreases compared to the PBS (*p* = 0.0327) and PP-3C group (*p* = 0.0629), respectively. In the onset cohort, the PP-pTau31 group showed 60.38% and 30.70% decreases compared to the PBS (*p* = 0.0465) and PP-3C groups, respectively. **(d)** IHC staining of astrocytes in the hippocampus of TauP301S mice brain after administration of vaccines. **(e, f)** Quantification of astrocytes signal stained by GFAP antibody in the hippocampus of TauP301S mice in the premorbid and onset cohorts. The results are expressed as IOD/area. In the premorbid cohort, the PP-pTau31 group showed 77.44% and 57.44% decreases compared to the PBS and PP-3C groups, respectively. In the onset cohort, the PP-pTau31 group showed 45.06% and 37.22% decrease compared to the PBS (*p* = 0.0287) and PP-3C groups, respectively. (**g**) IHC staining of pTauS396 in the hippocampus of TauP301S mice brain after administration of vaccines. Scale bar: 0.2 mm. (**h, i**) Quantification of pTauS396 signal stained by PHF13 antibody in the hippocampus of TauP301S mice in premorbid and onset cohorts. The results are expressed as IOD/area. In the premorbid cohort, the PP-pTau31 group showed 43.78% and 35.83% decreases compared to the PBS and PP-3C groups, respectively. In the onset cohort, the PP-pTau31 group showed 64.92% and 18.96% decreases compared to the PBS (p = 0.0074) and PP-3C groups, respectively. (**j**) IHC staining of pTauS404 in the hippocampus of TauP301S mice brain after administration of vaccines. (**k, l**) Quantification of pTauS404 signal stained by pTauS404 antibody in the hippocampus of TauP301S mice in the premorbid and onset cohorts. The results are expressed as IOD/area. In the premorbid cohort, PP-pTau31 group showed 38.36% and 18.49% decreases compared to the PBS and PP-3C groups, respectively. In the onset cohort, the PP-pTau31 group showed 54.74% and 31.96% decreases compared to the PBS (p = 0.0096) and PP-3C groups, respectively. All data represent the mean ± SEM. **p* < 0.05.

**Figure S7**

**
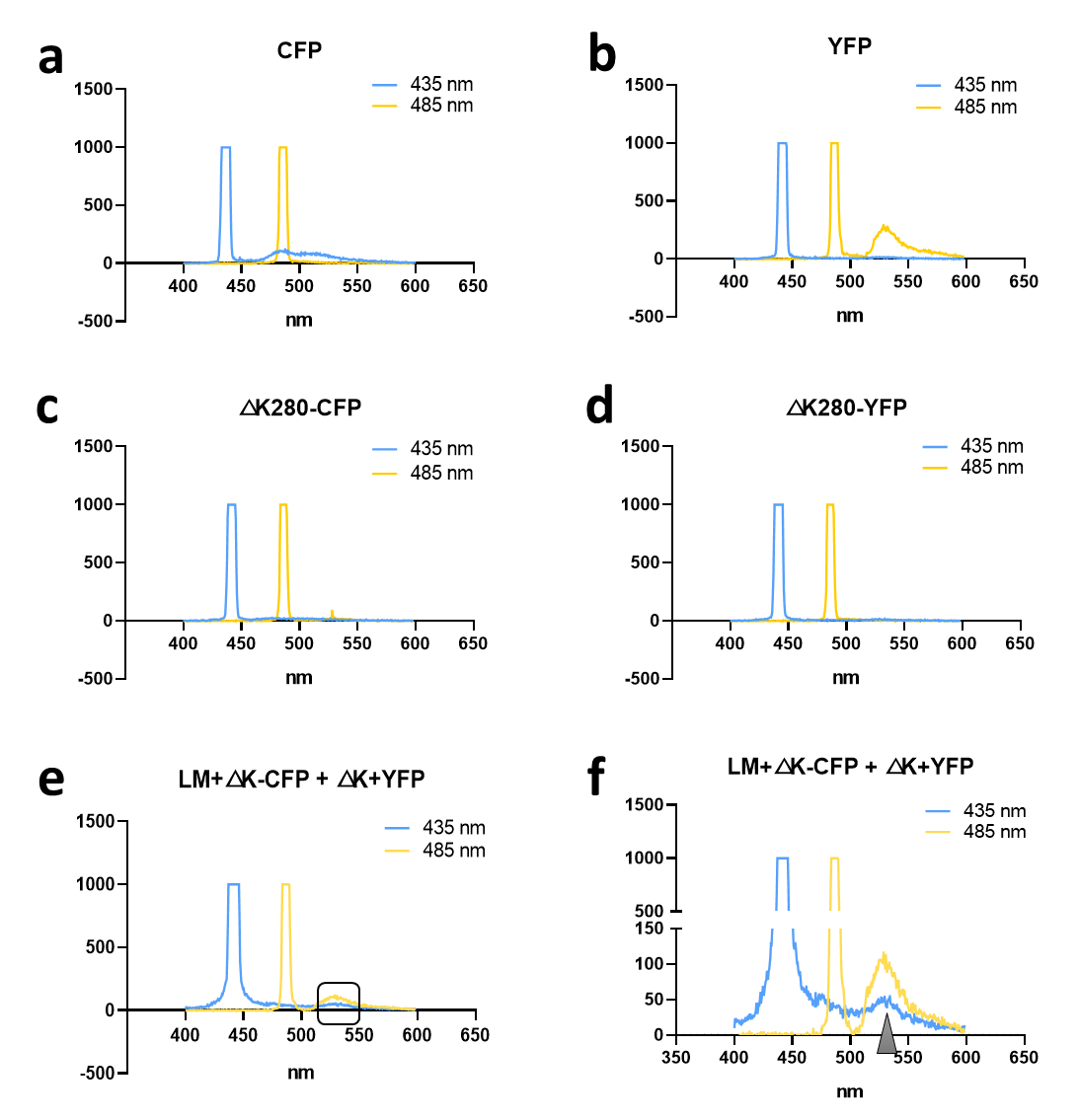
**

Fig. S7. The emission spectrum of plasmid-transfected cells under excitation of 435nm and 485nm. (a)-(d) show the emission spectrum of CFP, YFP, △K-CFP and △K-YFP plasmid-transfected cells under excitation of 435nm and 485nm, respectively. (e) shows the emission spectrum of 293T cell co-transfected with LM, △K-CFP and △K-YFP plasmid under excitation of 435nm and 485nm. (f) the enlarged view of the box in (e).

**Figure S8**

**
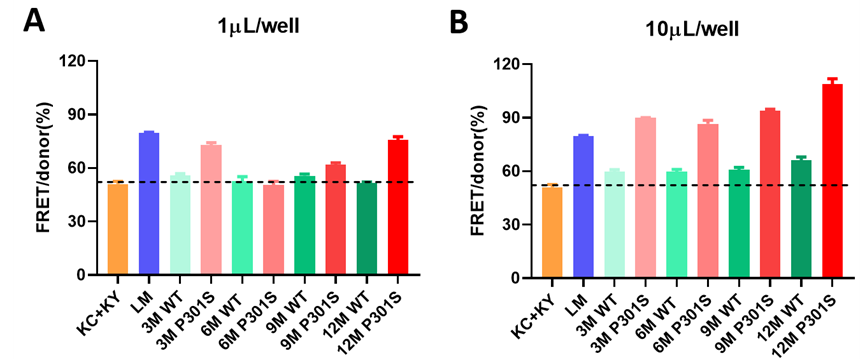
**

Fig. S8. The FRET signal induced by different age of mice. The FRET signal induced by 10 μL the RAB fractions of male wild mice (WT) or TauP301S transgenic mice (P301S) brain homogenate.

**Figure S9**


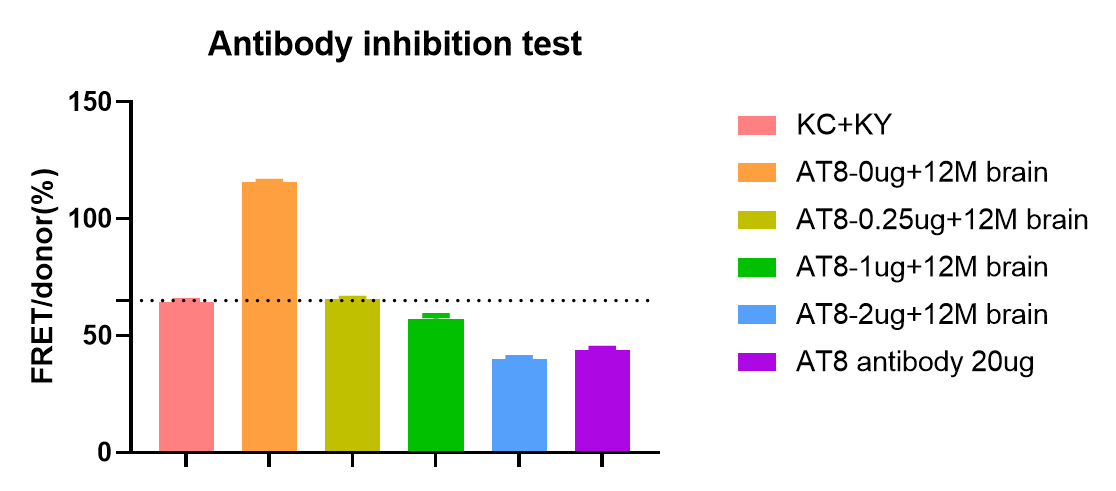


Fig. S9. The FRET signal of brain homogenate declined by inhibition from antibody. KY means △K-YFP, KY means △K-CFP, AT8 means mouse anti-pTauS202/T205 monoclonal antibody (Cat No. MN1020, Thermo Fisher Scientific, USA).

**Figure S10**


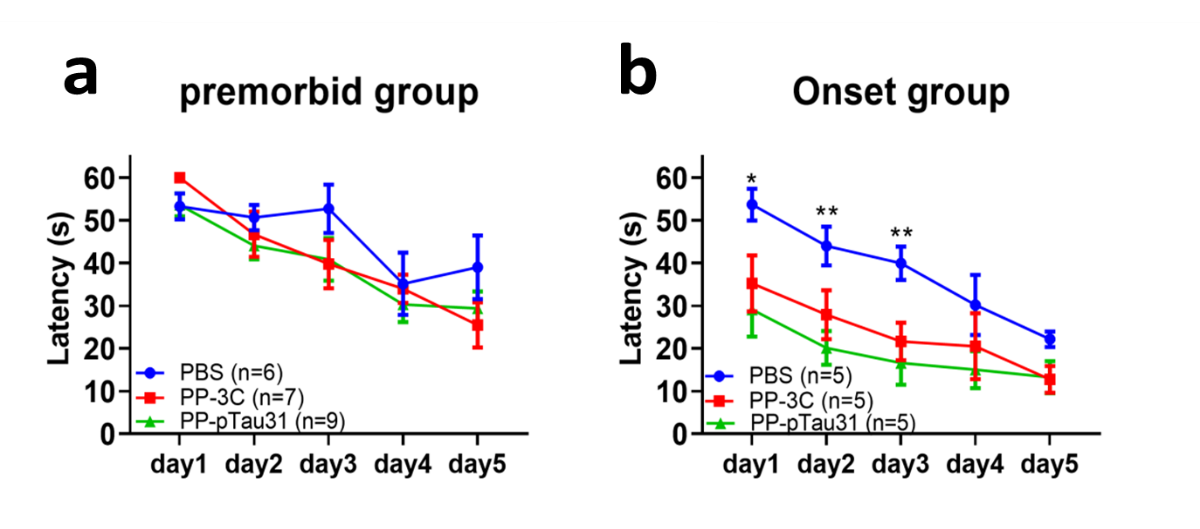


**Fig. S10. Latency of MWM in the premorbid cohort (a) and onset cohort (b).** All data represent the mean ± SEM. **p* < 0.05; ***p* < 0.01.

**Table S1. Sequences of the synthetic peptides designed in this study**

| Name | Phosphorylation site | Sequence | Original site |
| --- | --- | --- | --- |
| pTau30 | 202/205/238/262 | sspg**S**pg**T**pgsrksps**S**aksrskig**S**tenlC | [198-209]+[234-242]+[258-266] |
| pTau31 | 202/205/396/404 | sgysspg**S**pg**T**pgsrsrtpk**S**pvvsgdt**S**prC | [195-213]+[395-406] |
| pTau35 | 238/262/396/404 | ksps**S**aksrskig**S**tenlivyk**S**pvvsgdt**S**prhlC | [234-242]+[258-266]+[392-408] |
| Tau31 | 202/205/396/404 | sgysspgspgtpgsrsrtpkspvvsgdtsprC | [195-213]+[395-406] |
| pTau202/205 | 202/205 | spg**S**pg**S**pgsgggC-BSA | [199-208] |
| pTau238 | 238 | ksps**S**aksrggg-BSA | [234-242] |
| pTau262 | 262 | skig**S**tenlggg-BSA | [259-266] |
| pTau396 | 396 | ivyk**S**pvvsgggC-BSA | [392-400] |
| pTau404 | 404 | sgdt**S**prhgggC-BSA | [400-407] |

Two peptide sections were selected from human tau and spliced together without linker sequences. The residues lettered in upper case bold font type were phosphorylated, and their original phosphorylated sites in human total tau are shown.

**Table S2 Antibodies and reagents**

| Antibodies or reagents | Manufacturer | Catalog | Working concentration |
| --- | --- | --- | --- |
| Anti-pTau202/205 monoclonal antibody（AT8） | Thermo Fisher Scientific | MN1020 | WB（1：2000）, IHC (1：50) |
| Anti-pTau396 monoclonal antibody (PHF-13) | BioLegend | 829001 | WB（1：3000）, IHC (1：1000) |
| Anti-pTau404 polyclonal antibody | Thermo Fisher Scientific | 44-758G | WB（1：1000）, IHC (1：300) |
| Human Tau monoclonal antibody (HT7) | Thermo Fisher Scientific | MN1000 | WB（1：2000） |
| Anti-IBa1（EPR16588） | Abcam | ab178846 | WB（1：3000）, IHC (1：1000) |
| Anti-GFAP monoclonal antibody（2E1.E9） | Biolegend | 644702 | WB（1：3000）, IHC (1：1000) |
| GAPDH monoclonal antibody | Proteintech | 60004-1-lg | WB（1：10000） |
| Anti-HIS monoclonal antibody | Proteintech | 66005-1-lg | WB（1：5000） |
| HRP labeled goat anti-mouse monoclonal antibody | Jackson ImmunoResearch | 115-035-003 | WB (1:10000) |
| HRP labeled goat anti-rabbit monoclonal antibody | Jackson ImmunoResearch | 111-035-144 | WB (1:10000) |
| ECL solution | Dalian Meilun Biotechnology | MA0186 |  |
| Mouse monoclonal antibody isotyping reagents | SIGMA-ALDRICH | ISO2 |  |
| UltraVision Quanto Detection System HRP DAB | Thermo Fisher Scientific | TL-125-QHD |  |
